# Forward-scatter and side-scatter dataset for epithelial cells from touch samples analyzed by flow cytometry

**DOI:** 10.1016/j.dib.2015.12.027

**Published:** 2015-12-19

**Authors:** Cristina E. Stanciu, Ye Jin Kwon, Christopher J. Ehrhardt

**Affiliations:** Department of Forensic Science, Virginia Commonwealth University, USA

**Keywords:** Flow cytometry, Forensic science, Epithelial cells, Touch DNA

## Abstract

‘Touch’ or trace biological samples submitted to caseworking labs as evidence often contain biological material from multiple individuals which can result in mixed DNA profiles. These mixture profiles are difficult to interpret and may cause analytical bottlenecks for forensic laboratories. The data in this brief reports the variation in the relative abundance of intact epithelial cells deposited by four different donors across nine days. Touch samples were generated each day by rubbing a polypropylene tube with both hands for five minutes. Forward-scatter area (FSC-A) and side-scatter area (SSC-A) data was acquired with the BD FACSCanto™ II Analyzer. The relative abundance of different sub-populations within the FSC-A and SSC-A plots was calculated against the total number of events analyzed in each sample. Mean and standard deviation values were calculated for each donor.

**Specifications Table**

**Table:** 

Subject area	Forensic Biology
More specific subject area	Human Identification
Type of data	Table, Figure (optical scatter plots)
How data was acquired	Flow Cytometer (BD FACSCanto™ II Analyzer; FSC 150V, SSC 200V)
Data format	Analyzed
Experimental factors	Cell suspension was passed through 100 µm mesh filter prior to flow cytometry analysis
Experimental features	Four different donors each handled a plastic tube by rubbing with both hands for five minutes. The samples were collected with swabs, eluted in water, and analyzed with flow cytometry.
Data source location	Not applicable
Data accessibility	Data is in this article

**Value of the data**

• Data on the types and abundance of epithelial cells deposited onto a surface across individuals can elucidate transfer mechanisms and persistence of biological material on forensically relevant substrates.
• Differences in cell populations between individuals may be used to optimize and/or develop front end methods for separating cells prior to DNA extraction and typing, thereby simplifying mixture interpretation.
• Flow cytometry data may be used to study intrinsic variability in cell yields between individuals and investigate forensically relevant phenomenon such as ‘shedder status’ as they relate to DNA content in trace biological samples
• Abundance data may be used to optimize collection methods and evaluate effectiveness of existing sampling techniques for touch surfaces.

## 1 Data

Flow cytometry analysis was performed on epithelial cell populations from four different individuals across nine different sampling days. The relative abundance of cell events from the fraction containing larger forward scatter values was calculated against the total number of cell events analyzed in each sample. Mean and standard deviation values across all sampling days are tabulated for each individual. Optical data (forward scatter and side scatter plots in supplementary document) is summarized below.

**Table:** 

	**Percentage of cell events**					
**D02**	**D11**	**J16**	**E14**
**29-Oct**	21.31	26.28	5.06	5.11		
**31-Oct**	59.31	13.37	18.38	12.02		
**4-Nov**	26.72	35.87	7.30	8.86		
**6-Nov**	76.98	6.79	8.90	5.80		
**11-Nov**	43.34	30.99	ND	9.71		
**12-Nov**	20.75	16.65	11.61	7.80		
**2-Dec**	37.65	28.12	20.87	10.22		
**3-Dec**	38.34	29.13	32.36	13.22		
**4-Dec**	38.03	30.55	21.50	10.11		
						
**Mean:**	40.27	24.19	15.75	9.21		
**StDev:**	18.23	9.64	9.19	2.66		
**Student’s *t* test:** (*p* value)	**D02 v. D11**	**D11 v. J16**	**J16 v. E14**	**E14 v. D02**	**E14 v. D11**	**J16 v. D02**
	0.04	0.08	0.09	0.00	0.00	0.00

## 2 Experimental design, materials and methods

Touch epithelial cell samples were collected from volunteers using VCU-IRB approved protocol # ID# HM20000454_CR. Four volunteers (D02, D11, J16 and E14) were asked to rub a sterile conical tube (Cat#: 229421; Celltreat Scientific) in both hands for 5 min. Cells were collected from the surface with six sterile, pre-wetted swabs (22037924; Fisher Scientific) followed by two dry swabs. To elute the cells into solution, the swabs were manually stirred then vortexed for 15 s in 10 mL of Sterile DNAse-Free, Protease-Free Water (BP24701; Fisher Scientific).

The cell suspension was passed through a 100 µm mesh filter prior to Flow Cytometry Analysis on the BD FACSCanto™ II analyzer (Becton Dickinson) using 488 ηm and 633 ηm lasers and channel voltages of 150 V for FSC, and 200 V for SSC. Data acquisition was performed using the FACSDIVA Software (Becton Dickinson) with a stopping gate of 10,000 total events. The data was analyzed in FCSExpress 4.0 (DeNovo) by drawing a gate to include the large cell events in the sample, and exclude the debris population (see Supplementary figure). The relative abundance of large cell events was calculated against the total number of events analyzed in each sample. Mean and standard deviation values were calculated for each donor. The *p*-values for every donor combination were calculated using a two-sample of unequal variance Student’s *t* test.

