# Supplementary figures and images for: Forward-scatter and side-scatter dataset for epithelial cells from touch samples analyzed by flow cytometry
Source: Data Brief. 2015 Dec 19;6:416–8. doi: 10.1016/j.dib.2015.12.027 (PMC4709467; doi:10.1016/j.dib.2015.12.027)

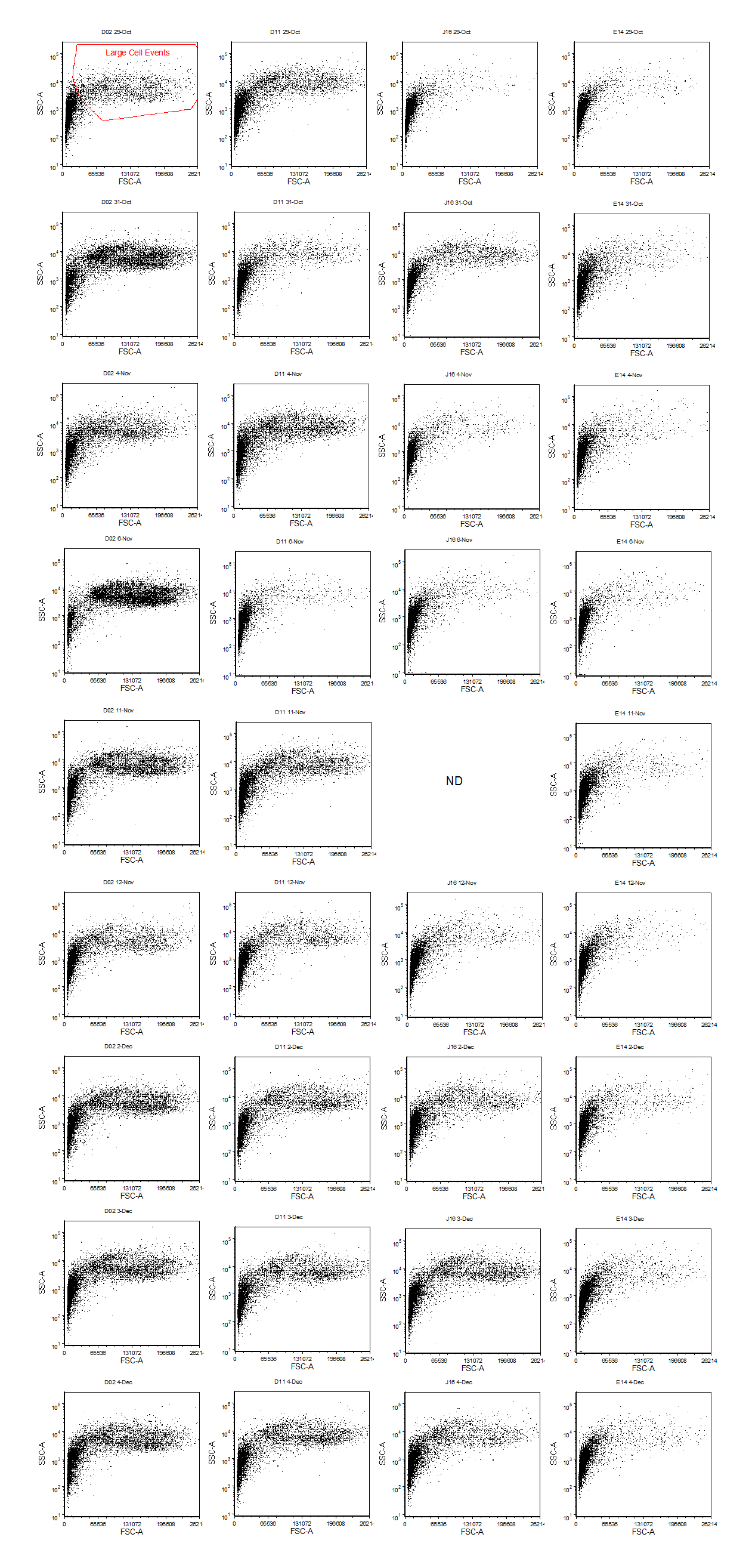

Supplement: Supplementary file 2 — Supplementary material [file mmc2.zip › FSC SSC Fig DiB compressed.tif]
